# Supplementary material for: Are carbon emissions trading and green financial instruments synergistic? -Comprehensive quantitative research based on content analysis
Source: PLoS One. 2024 Mar 7;19(3):e0298601. doi: 10.1371/journal.pone.0298601 (PMC10919601; doi:10.1371/journal.pone.0298601)
Supplement: S1 Data — (ZIP) [file pone.0298601.s001.zip › Data/地级市Z_F工作报告.zip!/╦─┤¿/░═╓╨╩╨/└┤╫╘╣1⁄2╓┌║┼í╛┬φ┐╦╩2╛▌═°í┐_2015.docx]

各位代表：

　　现在，我代表市人民政府，向大会报告2014年工作，对今年工作提出建议，请各位代表审议，并请市政协委员提出意见。

　　2014年工作回顾

　　过去一年，在市委的坚强领导下，在市人大、市政协的监督支持下，市政府团结依靠全市人民，沉着应对各种挑战，深入实施“统筹城乡、追赶跨越、加快发展”战略，着力稳增长、打基础、抓产业、促改革、惠民生，全市经济社会发展取得新的成效。

　　经济实现持续增长。始终保持加快发展的定力，牢牢掌控投资关键作用、产业支撑作用和消费促进作用，以项目工作统揽全局，突出重点集中发力，务求实效逆势上行。积极研判和应对形势变化，精准推出工业、消费稳增长两个17条措施的“组合拳”，有效化解经济运行中的突出矛盾和问题，推动了经济持续增长。与上年相比，全市地区生产总值增长9%，地方公共财政收入增长20.8%，全社会固定资产投资增长30.2%，社会消费品零售总额增长12.8%，城镇居民人均可支配收入、农民人均纯收入分别增长10.3%和12.3%。在经济发展新常态下，地区生产总值、社会消费品零售总额、城乡居民收入三项主要指标未达到预期，但总体上处于合理区间，与全国全省走势一致。

　　产业培育加快向好。深入推进产业兴市，坚持产城一体、产村相融，突出项目带动、园区承载、基地扩展，主攻四大重点成长型工业、生态农业和现代服务业，着力提质增效，确保经济增长。工业止滑回升。新引进5亿元以上工业项目11个，新开工重点项目60个，海螺水泥等55个项目竣工投产。“退城入园”企业中的鑫锐齿轮、中兴纺织新厂形成产能，普瑞制药迁建有序推进。工业投资161.5亿元、增长38.5%，新增规模企业31户，规上工业增加值在一季度下降0.4%开局不利的情况下全年增长10.1%，实现了止滑回升。农业加快转型。新培育省级重点龙头企业5家、各类农民专合组织186个。新建万亩现代农业示范园区16个、万亩林下养殖示范片6个，新发展茶叶8.2万亩、核桃16.8万亩、巴药3.8万亩、千亩蔬菜基地11个。“巴食巴适”综合性农产品区域公用品牌在北京、上海、广州、武汉、西安、成都等一线城市成功推介，影响力进一步增强。深入实施创新驱动发展战略，新增国家高新技术企业4家，建设山地特色种业创新基地8个，新获授权专利242件。第三产业稳步提升。新培育限额以上流通企业106家，西部国际商贸城一、二期投入营运，盘兴物流园入驻企业67家，回风、望王山等城市综合体加快推进，新开工秦巴国际养生城、裕博食品批发城等项目。启动实施了电子商务产业发展规划，建立了巴中市特色农产品防伪溯源平台、巴中物流服务平台和旅游产品电子商务网站，我市生产企业和零售企业实现线上线下同步销售。着力开发生态、红色和乡村旅游，创建了诺水河、佛头山、巴灵台3个AAAA级旅游景区和光雾山、诺水河洞天2个省级旅游度假区，建成平昌红军石刻标语园，打造了平昌县城-驷马-元山、南江正直-长赤-红光-沙河等乡村旅游环线精品，举办了光雾山红叶节、通江银耳节和系列乡村文化旅游节等，我市创建为“四川省旅游标准化示范市”，全年旅游收入89.6亿元、增长35.3%。组织开展银政企对接活动，授信900亿元，存贷款突破1000亿元，新增信贷117亿元，实现直接融资88亿元。全市产业发展呈现出一产趋优、二产领跑、三产提升的向好态势。

　　发展基础明显改善。立足既弥补欠账又适度超前，抓好城镇化、基础设施和生态环境建设，夯实发展基础。新型城镇化提质提速。强化规划引领作用，完成了巴城旧城改造、地下空间综合利用、国家森林城市创建等专项规划和8个片区城市设计，8个省级试点镇总规、控规完善或编制，以及22个中心村、556个聚居点、10个“巴山新居”示范片规划，在全省率先实现市县全域规划。持续推进城市建设，巴城北环线通车，巴恩快速通道双向通行，西环线、回风北路、江南二环路首段、东门大桥和草坝街特色街区等竣工，龙泉路口下穿式车道投入使用，“五创联动”和“三违”治理取得新的成效。兴文新区主干道通车，市政管网全面完工，市规划馆、客运中心站、西片农贸市场建成投营，市第一人民医院等36个公共配套项目有力实施。恩阳新城建成恩阳大道路基，芦溪河大桥、城区外环线等建设进展顺利。通江、南江、平昌县的旧城改造和新区、新城建设深入推进，县域经济发展平台进一步扩展和夯实。加快重点镇建设，8个省级重点镇、9个市级重点镇有力推进。深化“巴山新居”建设，新启动建设中心村22个、聚居点313个，新改造农村土坯(危旧)房4.8万户。四级城镇体系继续拓展和完善，城镇化率提高1.35个百分点。基础设施建设有力推进。巴达铁路进入轨道铺设阶段，巴广渝高速、巴陕高速桃园段加快建设，国道347线通(江)水(宁寺)段、542线沙(咀)信(义)段改造完工，陈家山隧道通车。红鱼洞水库枢纽工程开工，湾潭河、天星桥水库建设正常推进，二郎庙水库枢纽工程完工，双桥水库下闸试蓄水，黄石盘水库项目建设建议书获国家发改委批复、可研编制顺利推进。完成292座小型水库除险加固，小农水项目取得新的实效，新增灌面6万亩，改善灌面9.5万亩。曾口500千伏、清江110千伏变电站竣工投用，西部巴中云谷基地项目落地，新开通4G通信基站450个，有线电视数字化率达90%。生态优势继续巩固扩大。巩固退耕还林52.6万亩、林业自然保护区4.7万公顷，生态公益林补偿面积达到364.4万亩，森林覆盖率提高到57.1%。完成13家规模畜禽养殖场污染治理，淘汰燃煤小锅炉15座，关闭落后产能企业4家，单位GDP能耗下降5.1%。

　　全面深化改革扎实开局。坚持问题导向，深入贯彻落实中央、省委省政府和市委的安排部署，出台了新一轮改革有关实施方案，大力推动一批条件成熟的改革举措落地。行政管理改革稳妥实施。完成市、县(区)政府机构改革，行政审批项目市级减至97项、县(区)级减至150项，启动了建立行政权力清单工作。经济体制改革坚实迈进。注册资本由实缴制改为认缴制，推行117项“先照后证”改革，简化和放宽企业住所、经营场所登记管制，新增市场主体1.7万户。建立了土地出让弹性年期制、工业低效用地退出机制、大宗工业用地分期出让制，试点探索集体经营性建设用地使用权流转，申报入库立项城乡建设用地增减挂钩项目60个，在全省率先实行耕地占补平衡指标异地有偿使用，公共资源交易“五化”模式得到省政府肯定。实施了市圣泉水务公司增资扩股，市国资经营公司发行首支企业债5亿元，5家企业在场外市场挂牌，1家企业启动新三板上市。社会领域改革扎实推进。“小升初”免试就近入学全面执行，县级公立医院药品加成全面取消，启动实施了省内分级诊疗和异地就医即时结算，城乡居民养老保险激励机制和丧葬补助金制度在全省率先建成。在恩阳区、南江县开展了安全生产监管体制改革试点。农村改革进一步深化。完成了农村土地承包经营权确权登记颁证航拍、数据录入等基础工作，颁发特色产业所有权证、产业基地用益物权证54个，启动农村小型水利工程产权改革试点，建立了市县乡三级林权交易平台，试行林权流转基准价，农村产权抵押融资贷款9.1亿元。农业转移人口落户城镇户籍限制进一步放宽。

　　招商引资富有成效。坚持更加积极主动扩大开放合作，抓紧抓实招商引资第一要事。成功举办重协区第十七次市长联席会，充分借力中外知名企业四川行、西博会和友好城市等平台，创新拓展招引方式，突出产业招商、成链招商，引进重大产业项目65个，新落地项目188个，竣工项目115个，全年招商引资到位资金359.8亿元、同比增长25.6%。2011年以来，全市到位招商资金903亿元，预计到今年上半年，就可以提前完成市第三次党代会确定的五年到位招商资金1000亿元的目标。

　　民生保障切实加强。切实担当民生政府责任，把保障和改善民生作为发展的动力和目的，加快提升群众生产生活条件和水平。民生项目成效显著。全年民生项目财政投入140多亿元。建设保障性住房8180套、棚户区改造安置房4.37万户。新增城镇就业3.5万人。新启动实施15个片区连片扶贫开发，完成64个村的整村推进新村扶贫，全市新减贫人口11.36万人。实施通村通畅工程998公里、改善工程247公里、农村道路安保工程1033公里，新解决农村20.9万人安全饮水问题。巴城“菜篮子”蔬菜面积新增1.7万亩。城乡居民养老保险覆盖105万人，城乡居民基本医疗保险参保率达97.3%，城乡最低生活保障线标准分别提高到350元/月、2220元/年，城乡困难群众医疗救助金额达到1.23亿元，市残疾人康复中心主体工程、托养中心建成。办理法律援助案件2185件。社会事业发展给力。新建和改造校舍24.2万平方米、城区学校10所和公办幼儿园25所，组建了巴师附小、巴中中学等教育集团，南江小河职中创建为全国中职改革发展示范校。新引进社会资金兴办各类医疗机构19家，实施了36个乡镇卫生院规范化建设、314个村卫生室标准化建设，市中心医院升三甲、市中医院升三乙、3家中医院升二甲通过评审。南龛、佛头山等文化产业园加快推进，四级公共文化服务体系基本建成，文化遗产保护进一步加强，11件作品获得国家和省级大奖，文艺创作位居全省第一方阵。市体育馆、游泳馆主体工程完工，新建了60个农民体育健身工程，承办了国际公路自行车赛巴中段比赛活动。积极落实单独二孩政策，人口自然增长率3.74‰。社会治理持续强化。进一步规范乡镇便民服务中心运行，积极推行社区“扁平化”管理，扎实推进“法治巴中”建设，深入开展“六五”普法，依法化解了一批老难信访案件，安全生产、食品药品安全、治安管理、应急处突、打击违法犯罪和防灾减灾等工作责任落实、措施有力有效，全市没有发生重大事故和不稳定事件。其他事业和工作也取得了新进展、新成果，社会全面发展进步。

　　一年来，市政府主动适应全面从严治党新要求和经济发展新常态，高度重视加强政府自身建设，特别注重履行经济发展、公共服务、环境保护等职能，集中力量管理市场和社会能力不及的领域;特别注重依法行政，高度重视办理人大代表议案和政协委员提案，进一步完善了决策程序和机制，坚持用法治手段解决问题和矛盾，以制度建设为抓手，加强对行政权力的监督和制约，扎紧制度的笼子;特别注重转变作风，扎实开展党的群众路线教育实践活动，深入查找“四风”方面存在的突出问题，逐一制订、落实整改方案，立行立改，政风、行风和干部作风明显好转。

　　各位代表，过去一年发展改革稳定的成绩来之不易。我代表市人民政府，向全市广大干部群众，向驻巴部队、武警官兵和政法干警，向各民主党派、工商联、无党派人士、各人民团体，向所有关心和支持巴中发展的同志们、朋友们表示衷心的感谢!

　　全面回顾过去一年的工作，我们也清醒地看到：工业增长基础不稳固拖累经济发展的问题依然突出，传统消费需求基本饱和、新的消费热点尚未形成等因素导致消费短期内难以回升提振，部分重点项目要素保障难度加大影响了投资达到预期。扶贫开发和改善民生等任务仍然艰巨。少数政府工作人员适应经济发展新常态和作风建设新要求的能力不强，担当精神不足，干事创业激情减弱等。我们将正视这些问题和差距，努力解决这些问题，决不辜负全市人民的期望!

　　2015年工作安排

　　2015年是“十二五”规划收官之年，是实现市第三次党代会确定的五个翻番目标决战决胜之年。我们既面临经济高位增长、项目投资支撑、产业转型升级、要素资源保障的压力，同时也面临重点领域改革深入推进、社会资源市场配置开放合作空间扩大、国家出台定向支持政策、城镇化工业化双加速的历史机遇。今年，政府工作的总体要求是：深入贯彻党的十八届四中全会、省委十届五次全会和市委三届九次全会精神，全面落实中央、省委经济工作会议和市委的决策部署，主动适应经济发展新常态，抢抓发展新机遇，坚持稳中求进、又好又快，以项目工作统揽经济社会发展全局，以改革创新增强发展动力，以扩大开放激发市场活力，突出稳增长，着力扩内需、兴产业、惠民生，实现速度与质量并重、规模与效益均衡，保持经济中高速增长，促进社会和谐稳定。主要预期目标是：地区生产总值增长8.5%左右，全社会固定资产投资增长20%，地方公共财政收入增长15%，社会消费品零售总额增长13%，城镇居民人均可支配收入增长11.5%，农民人均纯收入增长14%，城镇登记失业率控制在4.3%以内，人口自然增长率控制在5.6‰以内，森林覆盖率达到57.5%，完成节能减排任务。

　　在当前形势下，全面完成上述预期目标不是一件容易的事，全市上下必须凝心聚力，攻坚克难，抓住重点，拼搏实干，为全面实现各项目标而倍加努力，奋力夺取经济社会发展的新胜利。

　　一、坚持以项目为统揽，保持投资高位增长

　　深刻把握新常态下经济增长更多依靠内需拉动的趋势性变化，充分挖掘城乡建设等领域投资潜力，使经济增长建立在投资持续扩大的稳固基础之上。

　　全力推进项目工作。项目是投资的主要抓手。实施重点项目248个，其中基础设施项目91个、产业发展项目109个、民生及社会事业项目48个，投资预期709亿元。严格落实领导和部门挂包责任，强化挂图作战、倒排工期、责任倒查等措施，年内新开工重点项目105个，新竣工重点项目57个。同时抢抓中央、省制定“十三五”规划的机遇，积极争取一批打基础利长远的项目进入国家和省的“盘子”。

　　加强基础设施建设。基础设施是吸纳投资的大容器，必须加力加快掀起新一轮建设高潮。交通方面，开工巴中机场主体工程、广巴铁路电气化改造、巴万高速，加快巴广渝高速、巴陕高速桃园段建设，建成巴达铁路，实施干线公路、农村公路建设等20个重点交通项目，抓好城乡公共交通基础设施建设。协同推进汉巴渝快铁、巴南城际客专和巴苍、镇巴渝高速公路前期工作。水利方面，加快红鱼洞水库枢纽工程、湾潭河水库大坝工程和巴城二期堤防及闸坝等城区防洪工程，基本完成天星桥水库枢纽工程，实施好小农水项目。力争黄石盘水库可研获得批复，江家口等2座大型水库、官房沟等5座中型水库纳入国家“十三五”规划。能源通信方面，完成张公220千伏变电站主线路架设、天然气分布式能源主要站点建设，加快“智慧巴中”建设。

　　加快推进城镇化。城镇化创造投资和消费需求，连着发展与民生，必须往深里推、往实里做，今年力争城镇化率提高2个百分点。扎实做好规划，完成城市总规、土地利用总规修编和城市控规、各专项规划编制，以及第三批4个省级试点镇总规、控规和中心村、聚居点规划编制，启动“多规合一”编制试点。加强“一城两翼”建设。巴城重点推进北环线东段、南环线建设和北环线、西环线配套联接工程，建成中杨干道，抓好江南二环路后续工程和西客站建设，积极实施江北客运站搬迁，启动国家园林城市创建，规划建设莲花山至鹰嘴山山地公园。扎实推进“五创联动”，争取获得全国文明城市提名城市表彰。兴文新区重点全面建成主干道，基本完工供水、供电、供气等工程，学校、医院、星级酒店等配套设施投入使用，初步建成西片区3平方公里核心区城市骨架，开工市体育中心、湿地公园，加快乐湾首府等3条商业街打造，抓好青年创业孵化园建设。恩阳新城重点完成巴恩快速通道、恩阳大道建设，启动义阳大道等城市道路和城区防洪堤、闸桥工程，加快置信产城一体示范园等重点工程，抓好曾家坝新城区建设，初步形成新城框架。推进县城改造和拓展。通江、南江、平昌县要继续加大旧城改造力度，加快高明新区、红塔新区、黄金新城、金宝新区以及城市道路、桥梁等市政设施建设，做大县城规模，增强吸纳经济和人口的能力。深化小城镇建设，立足就地就近城镇化，着力完善功能、提升品质，大力推进9个全国重点镇、12个省级试点镇建设，新规划和建设一批特色小城镇。深入实施巴山新居工程。坚持整体规划、产村相融、功能配套、注重实效，启动建设中心村21个、聚居点300个，改造农村危旧(土坯)房4.69万户，加快建设幸福美丽新村。

　　二、实施产业攻坚行动，做强经济发展支撑

　　深入推进产业兴市战略，保持抓产业发展的取向不变、力度不减、措施不软，加快走出一条“生态支持、绿色崛起”的路子，逐步实现经济增长主要依靠产业发展。

　　坚持量增质优突破发展工业。抓住目前园区承载能力增强、落地项目增多、投产企业增加的利好时机，打一场工业发展攻坚仗，奋力加快工业化进程。继续抓好园区建设，认真实施园区总体规划，重点推进园区“八通一平”设施配套，建立完善信息咨询、物流配送等公共服务平台，新拓展园区面积3平方公里。着力打造经开区"51025"省级园区，年内实现"335"产业发展目标。突出抓好工业项目，开工安碧捷芦笋精加工、秦巴现代高科中药产业园等项目50个，加快实施旭升机械、渝青机械等项目30个，投产建丰林业、威奥垃圾发电等项目40个，促进海螺水泥、万马汽车、同凯能源等企业扩产达效，实现工业投资162亿元。下大力气抓好企业培育，制订四大重点成长型工业培育计划和专项方案，着力打造“行业领军”“行业龙头”“行业标杆”企业，完善市县领导挂联帮扶企业机制，新培育小微企业100户以上，净增规模企业40户，规上工业增加值增长10.8%左右。切实抓好工业化信息化深度融合，加快巴中云计算中心、兴文现代综合信息服务基地建设，启动经开区、平昌数字化园区建设，培育两化融合示范企业20家。

　　立足特色生态转型发展农业。按照“巴山新居+特色农业+生态旅游”的大农业发展思路，着力构建新型农业产业链，推动农业提速增效、农民增产增收。加快创建山区现代农业示范市。深入实施核桃、茶叶、巴药“三百工程”，大力发展林业经济，新建云顶-灵山茶叶产业等万亩现代农业示范园区10个，新发展核桃14万亩、茶叶10万亩、巴药12万亩，新增食用菌基地1万亩、工业原料林10万亩。培育壮大新型农业经营主体。新引进培育市级以上龙头企业20家，发展各类农民专合组织300个、家庭农(牧)场100个。提升农业品牌效应。积极开发富硒、绿色、有机农产品，新增“三品一标”农产品认证40个。扎实创建“全国质量强市示范城市”，建立市级农产品质量安全追溯平台，启动建设川东北食品药品检验检测中心，完善“巴食巴适”综合性农产品区域公用品牌体系，着力打造“米仓山”核桃、“巴中云顶”茶叶、“巴药”等行业性公用品牌，精心组织“巴中产”“巴中造”产品展销，提升农业品牌形象和市场份额。增强农业综合生产能力。落实国家粮食安全战略要求，持续改善农业基础设施，新建高标准农田20万亩，粮食产量稳定在168万吨以上。

　　着力培育新的增长点提升服务业。服务业是我市培育新的经济增长点的潜力和希望所在，今年要努力实现发展提速、比重提高、水平提升。推进商贸物流业发展，加快西部国际商贸城三期、盘兴物流园、容邦太平洋购物中心等重点项目，抓好城区农贸市场、农村商贸服务中心和商贸集镇建设，积极培育现代物流、电子商务、现代金融、健康养老等产业，开展购物节等地方性促销活动，支持改善型住房、私家车、信息服务等消费，创建省级现代服务业集聚区2个，新培育限上企业50个。大力发展旅游业。明确“大景区、大市场、大旅游”的主攻方向，坚持科学规划、资源整合、理顺体制、引资开发，把巴中建成省内一流、全国知名的山地运动休闲旅游目的地，加快光雾山-诺水河AAAAA级旅游景区创建和世界自然文化遗产申报，争取创建南龛文化产业园、长赤玉湖-正直长滩2个AAAA级景区，实施水宁寺佛龛村等39个全国重点乡村旅游村建设，推进南龛、佛头山、恩阳古镇等景城一体化建设。积极开发高山滑草滑雪、避暑休闲养生等旅游项目和山地干果、地道药材、高山野菜等旅游产品，加强景区基础设施配套和旅游促销。实现接待游客1500万人，旅游收入115亿元以上。加快发展金融业。争取引进设立各类金融机构6家，全市股份制商业银行达到3家，新增信贷100亿元，保费收入25亿元。

　　三、深化重点领域改革，激发市场主体活力

　　进一步鲜明市场化改革取向，纵深推进投融资、行政审批、农村等重点领域改革，全面激发经济发展活力和内生动力。

　　加快投融资机制改革。贯彻落实国家鼓励和引导民间投资健康发展“新36条”和42项实施细则，加快推出民间资本投资我市基础设施、保障性住房、棚户区危旧房改造、市政设施、金融、电信、医疗卫生、养老等领域的项目，抓好巴中籍成功人士返乡创业回引工程，新增市场主体2万户。全面落实企业投资自主权，除法律和政策规定之外，政府不再审批企业投资项目。试点推进中小企业发行私募债，筹建中小微企业融资担保公司，积极培育上市企业。加快农村信用社改制、农村商业银行组建工作，加强资本运作，实现直接融资80亿元以上。注重完善农村产权评估、资产抵押等中介服务，实现农村产权抵押融资贷款15亿元以上。大力推广公私合营(PPP)模式，继续做好20亿元企业债券、10亿元私募债和5亿元中期票据发行工作，出资设立政府控股参股的融资担保和再担保机构。

　　深化行政管理体制改革。全面落实“两集中、两到位”和并联审批，部门行政审批机构整体进驻政务中心集中审批、提供服务，部门政务服务窗口授权到位，构建以窗口为主导的行政审批运行机制，提高现场办结率。规范运行保留的行政许可项目，构建审管分离的行政审批机制，建立省、市、县三级联动审批服务机制，实现网上审批和相关服务。全面实行“先照后证”和“三证合一”，推进企业注册全程电子化，加快建立企业信用联合惩戒机制，加强事中事后监管。进一步明晰区级政府事权，深化“扩权强镇”试点，充分发挥基层政府主观能动性。

　　深入推进农村各项改革。抓好符合条件的乡改镇、镇改街道办、村改居工作。推行流动人口居住证制度，进一步深化统筹城乡的养老、医疗、救助等社会保障制度改革。抓好巴州区全国第二批农村改革试验区建设，完善城乡建设用地增减挂钩、利益返农机制，开展农民进城落户有偿退出土地承包经营权、宅基地使用权和集体收益分配权试点，全面完成土地承包经营权确权登记，颁证率达到50%以上，试点镇完成“八权一股”确权登记。开展集体经营性建设用地流转试点，推进农村林权和国有林场改革，加快市县(区)乡(镇)产权交易流转平台规范化建设，完善农村资产市场定价机制，积极探索和完善土地股份、信托、合作等有效流转模式，实现流转耕地15万亩、林地10万亩以上。

　　四、积极扩大开放合作，务实推进招商引资

　　新常态下，必须坚持以开放思维、全球视角和全局观念谋划推动发展，把更多精力放在“外”字上，变外为内，变自我发展为合作共赢，努力拓展发展新空间、争创新优势。

　　深化拓展开放合作领域。加强与川东北城市合作，携手推进广巴达铁路等基础设施一体化建设、天然气开发转化和旅游环线打造。加强与成渝经济区合作，共同推进机械制造、食品饮料等产业技术研发。加强与成渝、关天经济区合作，协同推进汉巴渝铁路、镇巴渝高速公路等前期工作。加强与珠三角、长三角、环渤海合作，积极承接生物医药、纺织服装等产业转移。加强与上海自贸区合作，促进投资、贸易便利化和扩大外贸出口，扶持四川兆润集团等企业开拓国际市场，力争外贸进出口1.8亿美元。

　　更加求真务实招商引资。招商引资始终是第一要事。扎实开展“产业招商突破年”活动，充分利用省持续五年开展“中外知名企业四川行”等平台，立足招强引优，围绕四大重点成长型工业、农产品加工、文化旅游等主导产业和达芙妮制鞋、万马汽车、兆润机械等骨干企业，大力开展产业招商、沿链招商，新引进投资超5亿元工业项目10个、超5亿元现代服务业项目5个和产值过亿元农产品加工龙头企业1家。进一步健全项目挂联、政务服务、要素保障和重点考核项目竣工投产达效等机制，提高意向项目签约率、签约项目落地率、落地项目竣工率。全年新竣工招商引资项目70个以上，到位资金380亿元以上。

　　五、坚持不懈改善民生，促进发展成果共享

　　改善民生是政府的最大责任，政府工作的出发点和落脚点都是不断提高人民群众的生活水平。要进一步鲜明民生政府取向，采取更加务实的举措，抓好群众最盼望、最关注、最急需的事情。

　　扎实推进精准扶贫攻坚。开展《巴中市扶贫开发规划(2011-2020年)》中期评估，启动编制“十三五”扶贫规划。新实施15个、提升5个片区的连片扶贫开发，抓好40个整村推进项目。着力精准扶贫十项重点工作，新建农村廉租房2100余套，创建旅游扶贫精品示范村10个，打造扶贫互助社升级版30个，实施农村实用技术培训10万人次，每个贫困村至少新培育新型经营主体1个。对贫困人口登记造册，建立台账，做细做实，责任到人。年初建账，年底销账，全年新减贫人口10万人以上。

　　办好民生大事实事。认真实施省“十项民生工程”、20件民生大事和市“十件民生实事”。开工公租房4200套，建设棚户区改造安置房3.3万套，竣工拆迁安置房308万平方米。新建乡村公路192公里，解决24万农村居民和4.5万农村学校师生饮水安全，改善30所农村义务教育薄弱学校基本办学条件，建设边远山区标准化村卫生室300个。新增巴城“菜篮子”蔬菜面积1.5万亩。抓好38个村环境连片整治试点示范建设，实施656个行政村“宽带乡村”工程，完成投资3.8亿元的农村电网改造升级。

　　加强社会保障工作。大力创建省级创业型城市，开展大学生引领计划等专项行动，扶持2万名城乡劳动者成功创业，新增城镇就业2.7万人。深化医疗保险付费方式改革，异地就医和大病保险赔付即时结算率达到100%。启动实施全民参保登记计划，探索建立职工“五险合一”征缴机制，建立健全基本养老服务制度、残疾人社会保障和服务体系，完善城市“三无”人员、重特大疾病患者等困难群众临时生活救助政策措施，提高城乡低保和五保供养水平。切实保障妇女和未成年人权益，抓好留守学生(儿童)关爱工作，建成市社会福利院。积极开展灾害商业保险。

　　六、加快社会事业发展，提升公共服务水平

　　保障基本公共服务对统筹城乡发展、提高群众生活水平至关重要。必须大力加强社会事业发展，加快构建覆盖城乡的基本公共服务体系。

　　继续抓好教育事业发展。深入实施教育领域综合改革，鼓励集团化办学和社会资本参与办学，完善“四级职教体系”，促进公办教育与民办教育协调发展。推进城乡校点布局调整，大力改善办学条件，抓好巴中中学总部(兴文校区)、四川师大附属第四实验学校、恩阳中学分校、平昌金宝完全中学和通江高明职业技术学校等建设，完成巴中棠湖外语实验学校、巴师附小兴合小学建设和巴州区特教校高中部筹建。加大公立幼儿园投入，引导扶持民办幼儿园发展。建设农村教师周转房1100余套，建立农村教师待遇稳定增长机制，提升教师工作积极性和教学水平。

　　推进卫生计生事业发展。加快县级公立医院综合改革，大力支持社会资本办医。抓好重点卫生项目，建成市第一人民医院，开工市儿童医院、市精神卫生中心、巴中骨科医院兴文院区、平昌金宝医院等。加强基层医疗卫生机构标准化建设，以及人才队伍、医德医风建设。抓好20个基层计生服务站点示范建设，坚持计划生育基本国策，提高人口素质，促进人口健康均衡发展。

　　加快科技文化体育事业发展。实施创新驱动发展战略，深化科技体制改革，开展科技项目承包责任制试点，抓好“十百千万”科技创新工程，完成经开区科技企业孵化器一期工程，新增国家高新技术企业、省级创新型企业15家。开工市文化馆新馆，加快建设米仓古道、晏阳初等文化产业园和泉商文化展示中心，建成川陕革命根据地博物馆暨将帅碑林纪念馆新馆，推动重大文物保护项目实施，组建市文化投资有限公司，深入开展文化下乡活动。建成投运市体育馆、游泳馆，扎实推进山地运动项目“双千”工程，新建农民体育健身工程100个，承办好国际山地马拉松、国际公路自行车赛等综合性赛事，促进体育事业加快发展。

　　支持工会、共青团、妇联等开展工作，加快发展老龄、残疾人等事业，抓好档案、地方志、统计、民族、宗教、侨务、外事、对台、保密、防灾、地震、气象、人防等工作。开展全国双拥模范城创建，强化国防动员和民兵预备役建设，促进军地融合发展。

　　加强法治政府建设

　　各位代表，我们深知全面切实履行职能，建设法治政府非常重要。我们将坚定法律信仰，坚守“法在我心中”，坚持在法治的轨道上工作，始终做到依法行政、高效行政、廉洁行政。

　　切实规范行政职权。加快建立行政权力清单目录，6月底前完成行政职权目录、行政审批相关目录、公共资源交易目录清理、编制和公开发布工作，积极建立和推行负面清单、责任清单制度，建立健全政府购买公共服务机制，进一步厘清行政权力边界，做到法定职责必须为、法无授权不可为。

　　依法科学民主决策。建立政府常务会议学法制度，会前学法每月两次，专题学法每年两次。健全法定决策程序，严格执行法律顾问、行政机关内部重大决策合法性审查制度，加强规范性文件备案审查。积极发展政府协商民主，强化决策咨询，畅通民间智库发挥作用渠道。坚持“三重一大”决策提请市委审定，探索重要决策提前与市人大联系沟通和与市政协民主协商机制。

　　加强权力制约监督。建立行政决策终身责任追究制度、责任倒查制度。进一步规范行政裁量权，3月底前公开发布裁量权标准，试点推行跨部门综合执法，着力整改行政执法突出问题，积极探索权力集中部门和岗位分事行权、分岗设权、分级授权、定期轮岗。自觉接受人大法律监督、政协民主监督以及专项执法检查，支持法院审理行政案件、执行生效判决，保障依法独立行使审计监督权，拓宽舆论、社会和群众监督渠道。大力推进财政预算、公共资源配置、重大建设项目、社会公益事业等领域的政府信息公开，确保权力公开运行。

　　深化普法依法治理。深入开展“法律七进”和创建“依法行政示范单位”活动。坚持用法治方式化解土地征用、房屋拆迁、企业改制、环境保护等工作中引发的矛盾，切实抓好劳资医患纠纷调解、农民工维权、消费维权、青少年法制教育、涉法涉诉信访依法终结等法治惠民实事，加大食品药品安全、交通运输、农林水利、文化旅游、安全生产等重点领域执法力度，严厉打击各类违法犯罪活动，努力构建法治良序，促进社会和谐稳定。

　　各位代表，今年的政府工作繁重而艰巨，需要集思广益用好机遇加快发展，需要攻坚破难深化改革化解矛盾。我们恳请人大代表、政协委员和社会各界加强对政府工作的监督，支持和推动政府自身建设。让我们紧密团结在以习近平同志为总书记的党中央周围，按照“四个全面”的部署和要求，在市委的坚强领导下，奋力开创巴中改革发展的新局面，努力交出一份全市人民点赞的合格答卷!
